# Application of White-Wine-Pomace-Derived Ingredients in Extending Storage Stability of Fresh Pork Burgers

**DOI:** 10.3390/foods12244468

**Published:** 2023-12-13

**Authors:** María Jesús Martín-Mateos, Jonathan Delgado-Adámez, Daniel Moreno-Cardona, M. Esperanza Valdés-Sánchez, M. Rosario Ramírez-Bernabé

**Affiliations:** Technological Agri-Food Institute (INTAEX), Centro de Investigaciones Científicas y Tecnológicas de Extremadura (CICYTEX), 06071 Badajoz, Spain; mariajesus.martinmat@juntaex.es (M.J.M.-M.); jonathan.delgado@juntaex.es (J.D.-A.); daniel.moreno@juntaex.es (D.M.-C.); esperanza.valdes@juntaex.es (M.E.V.-S.)

**Keywords:** by-products, white wine pomace, storage stability, meat product, antioxidant, phenolic compounds

## Abstract

White wine pomace, a by-product from winemaking, was stabilized after the application of thermal blanching (with the aim of deactivating the polyphenoloxidase enzyme), milling, and processing by hydrostatic high-pressure treatment (with the aim of reducing initial microbial loads while preserving phenolic compounds content). The valorized pomace (VP) ingredient was added at different proportions to pork burgers (0.5%, 1%, and 3% *w*/*w*) to improve their preservation, and the effect was compared to those produced by sulfites and with a control (without sulfites or VP). Burgers were vacuum-packed and refrigerated for 7 days. Microbiological, color, oxidation, and sensory parameters were analyzed. Neither sulfites nor VP reduced the microbial development of most microorganism groups evaluated (*p* > 0.05); however, both prevented coliform growth during storage (*p* < 0.01). The use of sulfites prevented the discoloration of burgers during storage, while VP had no effect (*p* < 0.001). On the contrary, VP limited lipid and protein oxidation development during storage (*p* > 0.05), while sulfites had no effect. Therefore, the use of VP from white wine production could have an antioxidant effect but a limited antimicrobial or color-protective effect for the preservation of pork burgers.

## 1. Introduction

Microbial growth, lipid oxidation, and color are important factors that limit the shelf-life of meat. Lipid oxidation reduces the nutritional value of meat and meat products since it generates a series of chemical reactions that can alter physicochemical parameters and sensory attributes (odor, color, and flavor), causing a decrease in consumer acceptance [1]. To prevent these problems, the meat industry often adds food additives like sulfites. The use of sulfites in meat products is common in the meat product industry (Council Directive N° 95/2/EC of 20 February 1995), and they delay microbial spoilage and the discoloration resulting from myoglobin oxidation [2]. However, the presence of these additives in burgers can lead to consumer rejection due to their relation to the development of allergic and respiratory reactions and other health problems [3]. In addition, sulfites can produce the loss of the nutritional value of foods associated with reduced bioavailability of some vitamins, such as thiamine (B1), folic acid (B9), pyridoxine, and nicotinamide [4,5]. The effect of this synthetic additive could be replaced by other natural additives, so the meat industry is looking for new alternatives to produce healthier meat products, such as the replacement of additives with natural plant extracts [6].

Winemaking is one of the most important agro-industrial activities in several countries of Europe, and as a result, the productive system generates large amounts of by-products. The main by-product of winemaking is wine pomace, also called grape pomace, a solid by-product generated after grape pressing that contains mainly seeds, skins, stems, and remains of grape pulp.

The composition of wine pomace will differ depending on whether it is red or white wine pomace, not only due to the different chemical compositions of the grapes but also because of the type of vinification. In the red wine processing, juice and pomace are fermented together. However, in the white winemaking process, only the juice is fermented after pressing, and for this reason, pomace from white-wine-making processes have more pulp and residual sugar when compared to red wine pomace, so it generally contains more water [7,8]. These by-products represent waste disposal, are used for wine alcohol production, or serve as fertilizer or animal feed [8]. Nevertheless, wine pomace still contains a wide range of interesting compounds, such as dietary fiber, polyphenols, polysaccharides, fatty acids, and minerals [8,9], so it can be reutilized by the food industry. The antioxidant and antimicrobial capacities of the polyphenols present in grape by-products have been clearly demonstrated [10]. For this reason, phenolic compounds present in grape seed extracts and other food by-products may also have a protective effect against meat discoloration [9,11] since the link between pigment oxidation and lipid oxidation has already been stated [12]. Polyphenolic compounds exert strong antioxidant potential by, e.g., scavenger-free radicals and inhibiting enzymes responsible for their formation, and their antimicrobial effect is attributed to an alteration in morphological changes in microorganisms and the loss of integrity of the bacterial cell wall and its permeability [6]. Nevertheless, proteins, fats, and minerals present in meat also potentially interact with these active compounds and may lead to reduced antioxidant and antimicrobial activity [6], so wine pomace should be incorporated into meat products at an optimal level. In addition, immediately after production, grape pomace contains a microbiological load that could favor spoilage. Consequently, it is very important to stabilize the wine pomace microbiologically and enzymatically before adding it to meat products. Novel non-thermal technologies, like hydrostatic high pressure (HHP), allow for the integral use of the grape pomace in order to obtain an ingredient that could be added as a bioactive ingredient [13,14]. HHP consists of subjecting the food to pressures from 400 to 600 MPa during a short period of time at a room-refrigerated temperature. HHP is applied by introducing the food previously sealed in the final package to a vessel that is filled with water. Subsequently, water is pumped inside the vessel until it reaches a determined pressure. After a period of several seconds or minutes, the pressure is released instantaneously. The main advantage of the use of HHP for the valorization of grape pomace is that the process allows an integral use of the pomace (the whole by-product) so that no residues are generated, and no solvents are required; thus, it could be considered a green technology. However, the treatment did not deactivate enzymes, like the polyphenoloxidase of the pomace, which remains active during storage post-processing [13,14]. Hence, the aim of this work was to stabilize white grape pomace by HHP and to investigate the effect of the incorporation of three different levels of the valorized by-product on the microbiological, physicochemical, and sensory properties of pork burgers stored for 7 days and to compare it with the protective effect of sulfites. The valorization and application of white grape pomace have been scarcely studied compared to red wine pomace.

## 2. Materials and Methods

### 2.1. Material

#### 2.1.1. Valorization Process of the White Wine Pomace

White wine pomace (WWP) (cv *Cayetana*) was provided by a local wine manufacturer company located in Alburquerque (Badajoz, Spain) in September 2021. Approximately 10 kg of destemmed grapes were collected, and vacuum-packaged (1–2 kg) at −80 °C until being used. Since our previous studies [13,14] showed that HHP did not reduce polyphenoloxidase (PPO) activity, and as a consequence, phenolic compounds content was reduced during storage, the white wine pomace collected was thermally blanched before HHP. An Exhauster unit was used for the thermal treatment, applying steam at 103 °C. The unit was equipped with lower and upper stainless steel mesh belt with variable speed drag (1 min of residence). These conditions were decided based on our previous experiments in which scalding was applied at times of between 1–5 min, and then, the activity of PPO and the content of total phenols were evaluated. 

After the thermal blanching (TB), pomace was placed in trays and frozen in a chamber at −18 °C. Being well-frozen, the pomace was crushed in a Thermomix (Vorwerk, Germany) at maximum speed for 1–2 min. Pomace was crushed-successive freezing (3 cycles) until a fine powder was obtained. Once crushed, it was packed in vacuum bags and processed by HHP at 600 MPa for 5 min. For this purpose, the vacuum-packed pomace was placed in a vessel, and subsequently, water was pumped into the vessel until a pressure of 600 MPa was reached. After 5 min, the pressure was instantly released. The ground vacuum-packaged pomace was processed in semi-industrial Hiperbaric equipment (6000/55, Hiperbaric, S.A., Burgos, Spain) with a 55 L capacity container, and the initial temperature of the water was 16 °C. Processing conditions were chosen on basis of previous studies [13,14], which showed that the product could reach long time of stability after processing (at least 9 months at refrigeration), so it could be applied by meat products industry during the whole year despite this is a seasonal by-product. 

The ingredient (valorized pomace, VP) (obtained after TB, milling, and HHP; TB+HHP) from white wine pomace was vacuum-packaged and saved at −80 °C until their use for meat products manufacture. It was added in different proportions to pork burgers.

#### 2.1.2. Burgers Manufacture and Experimental Design

When preparing the burgers, a traditional formulation was followed. The minced pork meat (acquired in a local market) was mixed with the following ingredients: 13 g kg^−1^ of salt; 1.5 g kg^−1^ of dehydrated garlic, 1.5 g kg^−1^ dehydrated onion, and 0.5 g kg^−1^ black pepper. This formulation was used as a control (negative control without additives). A positive control (with additives) was prepared with the same formulation as before but also with sulfites (0.45 g kg^−1^) added (88394-Sodium disulfite, Guinama S.LU, La Pobla de Vallbona, Spain). The burgers with the ingredient of pomace were prepared with the same proportions of negative control (salt and spices), and the pomace ingredient was added in three growing levels (0.5, 1, 3%, *w*/*w*).

The levels of VP were chosen on basis of a previous experiment in which burgers with variable ranges of pomace (0–10% *w*/*w*) were prepared and tasted by trained panelists. The maximum level of pomace, which was not considered undesirable by panelists, was 3% (*w*/*w*).

Therefore, five formulations of burgers were manufactured: Control (burgers manufactured without sodium metabisulfite); MTB (burgers manufactured with sodium metabisulfite); VP-0.5 (burgers manufactured with 0.5% (*w*/*w*) valorized pomace); VP-1 (burgers manufactured with 1% (*w*/*w*) valorized pomace); VP-3 (burgers manufactured with 3% (*w*/*w*) valorized pomace). Burgers were analyzed on day 1 (the day after the manufacture) and after 7 days of refrigerated storage (6 ± 2 °C). All burgers were individually vacuum-packaged in plastic bags (polyamide polyethylene 20/100, oxygen permeability 50 cm^3^ m^−2^, 24 h^−1^ and 0% relative humidity, 120 µm thickness, Eurobag). Five burgers per batch (formulation x storage time) of 100 g each were manufactured; therefore, a total of 50 burgers were studied in the experiment (5 × 5 × 2).

### 2.2. Methods

#### 2.2.1. Polyphenoloxidase (PPO) Enzyme Activity in Pomace 

Analysis of the PPO activity in pomace extracts was carried out as described by González-Cebrino et al. [15] with some modifications. Absorbance was measured at 420 nm and 25 °C for 3 min in a Thermo Scientific Evolution 201 UV-Vis spectrophotometer (Thermo Scientific™, Fisher Scientific SL, Madrid, Spain) in kinetic model. The activity of the enzyme was expressed as a percentage of activity with respect to the control samples.

#### 2.2.2. Profile of Individual Phenolic Compounds of VP 

Polyphenolic substances of 25 g of VP were extracted with methanol/water/formic acid (50:48.5:1.5, v/v/v/v) according to the method described in Portu et al. [16] with slight modifications [17]. The phenolic extracts were filtered through PCX SPE cartridges; the catechins, flavonols, proanthocyanidins, phenolic acids and their esters and stilbenes eluted were concentrated on a rotary evaporator (40 °C) and redissolved in 20% (*v*/*v*) aqueous methanol solution. The phenolic substances were analyzed by HPLC by an Agilent 1200 LC system (Agilent Technologies, Palo Alto, CA, USA) equipped with a degasser, quaternary pump, column oven, 1290 infinity autosampler, UV-Vis diode-array detector (DAD), fluorescence spectrophotometer detector (FLD), and the Chemstation software (version A.02.10. [026]) package for LC 3D systems (Agilent Technologies, Palo Alto, CA, USA) to control the instrument and for data acquisition and analysis was used. Separation was performed in a Licrospher^®^ 100 RP-18 reversed-phase column (250 × 4.0 mm; 5 µm packing) with pre-column Licrospher^®^ 100 RP-18 (4 × 4 mm; 5 µm packing). Eluents used for the analysis were (A) acetonitrile/water/formic acid, (3:88.5:8.5, v/v/v), (B) acetonitrile/water/formic acid (50:41.5:8.5, v/v/v), and (C) methanol/water/formic acid (90:1.5:8.5, v/v/v). The column was maintained at 40 °C. All phenolic compounds were identified according to their elution order and retention times of the commercial standards [16,18]. For the identification and quantification of the compounds, chromatograms were recorded at 280, 320, 360, and 520 nm on the diode array detector (DAD) (+)-catechin and pro-cyanidin B2 were identified and quantified by FLD with excitation at 280 and emission at 320 nm. Calibration curves of their respective standards (R^2^ > 0.999) were used for the quantification and calibration of each compound [17]. Flavanols were quantified as mg of (+)-catechin·kg^−1^ WB, while flavonols as mg of quercetine-3-glucoside·kg^−1^ WB and phenolic acid as caffeic acid·kg^−1^ WB. Results are expressed as percentage with respect to the sum of total individual phenolic compounds identified in this study.

#### 2.2.3. Physicochemical Composition of Valorized Pomace and Burgers

The proximate composition: protein, fat, and moisture content were evaluated in the initial pomace and burgers. Moisture and protein were determined according to the AOAC (2016), and fat content was analyzed by Folch method [19,20]. Fiber content of grape pomace was determined according to the modified Southgate method [21]. pH was evaluated with a pHmeter Crison pH 25 + (Crison, Barcelona, Spain), and the water activity (aw) measurement was carried out at 25 °C using a Novasina Labmaster-aw meter (Novasina AG, Lachen, Switzerland), which offers temperature-controlled measurements. In addition, fatty acid methyl esters were analyzed using an Agilent 6890 gas chromatograph (Agilent Technologies, Santa Clara, CA, USA) equipped with a flame ionization detector (FID) and a fused silica column (60 mm length, 0.25 mm inner diameter and 0.25 m film thickness). The injector and detector temperatures were 260 °C and 280 °C, respectively. Column oven temperature was raised to 220 °C on a ramped temperature, and helium was used as a carrier gas with a constant flow of 1.2 mL min^−1^ and make-up of 25 mL min^−1^. Injection mode was used with a split ratio of 1:100. Individual FAME identification was carried out on the basis of Sigma standards (Supelco 37 component FAME mix standard, Sigma Aldrich, St. Louis, MO, USA) compared with the retention times obtained. Results are expressed as a percentage of total fatty acid methyl esters.

#### 2.2.4. Microbiological Analysis of Valorized Pomace and Burgers

Firstly, 10 g of sample were taken aseptically and homogenized with 90 mL of sterile peptone water in a laboratory blender (Stomacher R_ 400 Circulator) for 1 min. Serial decimal dilutions were prepared in sterile peptone water, and 1 mL of each sample was spread on suitable culture media. Mesophilic aerobic bacterial counts were performed using a standard Plate Count Agar (Merck, Darmstadt, Germany, 1.07881), and plates were incubated at 30 °C for 72 h. Psychrophilic counts were determined on Plate Count Agar (Merck, 1.07881) after incubation at 7 °C for 10 days; molds and yeasts were incubated on Potato Dextrose Agar at 25 °C for 5 days; *Staphylococcus aureus* were determined on Baird Parker Agar (Merck, 1.05406) after incubation at 37 °C for 24–48 h; sulfite-reducing bacteria were incubated on Tryptose Sulfite Cycloserine Agar (Merck, 1.10235) at 37 °C during 24 h; *Enterobacteriaceae* were determined on Violet Red Bile Glucose Agar (Merck, 110275) at 37 °C for 24–48 h, and total coliforms and *E. coli* were incubated on Chromocult Agar (Merck, 1.10426) at 37 °C for 24–48 h. Finally, *Salmonella* spp. and *Listeria monocytogenes* were determined following the ISO 6579-1, 2017 [22] and ISO 11290-1, 2017 [23], respectively. Results were expressed as Log10 CFU (colony forming units) g^−1^. The detection limit of the above techniques was 10 CFU g^−1^. 

#### 2.2.5. Instrumental Color of Burgers

Instrumental color determinations were performed with a Minolta CM-5 spectrophotometer (Minolta Camera, Osaka, Japan). The color coordinates of lightness (L*), redness (a* red/green axis), and yellowness (b* yellow/blue axis) in the CIE Lab color space were analyzed. In addition, hue angle was calculated (h°= tan^−1^ (b*/a*)) as well as the saturation index or Chroma (C*) (C = (a^* 2^ + b^* 2^) ^0.5^). Two readings were recorded (one for each side of the burger), and the mean of the two readings was obtained.

#### 2.2.6. Lipid and Protein Oxidation of Burgers

Lipid oxidation was assessed by thiobarbituric acid reactive substances (TBA-RS) according to Sørensen and Jørgensen’s method [24]. TBA-RS values were calculated from the standard (1,1,1,3-tetraethoxypropane, TEP) curve, and the results were expressed as mg of malondialdehyde per kg of sample (mg MDA kg^−1^). Protein oxidation was assessed by measuring the carbonyl groups formed during incubation with 2,4-dinitrophenylhydrazine (DNPH) in 2 N HCl following the method described by Oliver et al. [25]. The absorbance measurement of protein concentration was detected at *λ*-280 nm spectrophotometry using a spectrophotometer (Evolution 201 UV-Visible, Thermo Scientific) with bovine serum albumin (BSA) as standard. Protein oxidation was expressed as nmol carbonyls mg protein^−1^.

#### 2.2.7. Total Phenolic Compounds Content in Pomace and Burgers

The levels of total phenolic compounds in the initial pomace and burgers were determined by the Folin–Ciocalteu colorimetric method [26]. Gallic acid was used for the generation of a standard curve, and the results were expressed as milligrams of Gallic acid equivalent (GAE) per 100 g of sample weight on wet base (WB).

#### 2.2.8. Sensory Analysis of Burgers

For the sensory analysis, an independent assay was replicated with 5 burgers per batch (5 × 4). Only burgers on day 1 were tasted because of panelists’ safety. Cooked burgers were evaluated by 8 trained individuals selected from the staff members of CICYTEX. All the panelists were experienced in sensory evaluation of meat products. The analyses were performed in individual booths under white light. Panelists were provided with water to help eliminate any aftertaste between samples.

The burgers were cooked on a double electric grill at an internal temperature of 72–75 °C and then were cut (2 × 2 cm^2^), wrapped in aluminium foil, and stored in thermal boxes for temperature conservation until presentation to the tasters. Each panelist was provided with a sample of burger from each batch that was individually coded with three-digit random numbers. A descriptive analysis was carried out. The following descriptors were evaluated: general appearance, odor intensity, intensity taste, texture, unpleasant taste, and general evaluation. The intensity of each parameter was evaluated on a lineal, 10 cm non-structured scale from 0 (I do not like it) to 10 (I like it very much).

### 2.3. Statistical Analysis

Three samples of pomace (*n* = 3) and five burgers (*n* = 5) per treatment were analyzed. In order to evaluate the changes during processing in grape pomace (Table 1), a Student’s *t*-test was applied between initial pomace vs. TB pomace (*p*-value initial-TB); another Student’s *t*-test was applied between TB vs. HHP pomace (*p*-value TB-HHP); finally, an ANOVA test was applied to evaluate differences among the three groups (*p*-value treatments; and then a Tukey’s HSD test was applied when differences were significant). For the experiments with burgers, one-way analysis of variance (ANOVA) was performed to analyze the effect of the formulation; in addition, another one-way ANOVA was applied to evaluate the changes during storage (day 1 vs. day 7). HSD Tukey’s test was applied to compare the mean values when ANOVA showed significant differences due to the effect of burger formulation. SPSS, Version 21.0 (SPSS Inc., Chicago, IL, USA) was utilized. Data were analyzed at three levels of significance (ns: *p* > 0.05; * *p* < 0.05; ** *p* < 0.01; *** *p* < 0.001). Mean values with standard deviation are reported.

## 3. Results and Discussion

### 3.1. Manufacture Process of the Ingredient of White Wine Pomace (VP) and Characterization

In the WWP, the PPO activity was significantly reduced after the thermal blanching (TB), with a 99% decrease from the initial level, and then, low levels of activity also remained after HHP (1.3%) (Table 1). On the other hand, the levels of phenolic compound content (PPC) significantly increased after TB. Blanching is a heat treatment usually applied on vegetables prior to food processing to preserve the product quality during long-term storage because it deactivates the enzymes and destroys microorganisms that might contaminate them [27]. Several studies have shown that heat pretreatment favors the release of phenolic compounds from vegetables and skins [27,28,29], which could be attributed to the structural changes induced in the cellular matrices. The blanching process helps maintain the stability of phenolic compounds after HHP by deactivating the PPO enzyme. In addition, the HHP was able to provide microbial stability to the WWP since the treatment applied was able to achieve a significant reduction in all microorganisms analyzed. Concretely, mesophilic counts were significantly reduced after HHP, while the TB produced an intermediate reduction with respect to initial counts. The mold and yeast counts were significantly reduced after TB and HHP. A similar trend was found for *Enterobacteria*, and counts were under the detection limit after TB and HHP. 

Previous studies on red and white grape pomace treated by HHP have shown that the treatment achieved a sufficient deactivation of microorganisms to reach a shelf-life of at least 9 months [13,14]. This long shelf-life would allow for preserving the WWP from one year to the following since it is a seasonal by-product. On the other hand, the need to deactivate the PPO enzyme to preserve the phenolic compounds of the treated pomace during storage was relevant. This is an important concern for the valorization of pomace since phenolic compounds are the main bioactive compounds of pomace, and they have antioxidant and antimicrobial activity [1,8], so their preservation is essential to maintain their bioactivity.

In the current study, the application of a TB prior to HHP of the pomace would allow for obtaining a stable product from an enzymatic point of view (thanks to the effect of TB on the PPO enzyme). At the conditions of TB application, the reduction in PPO was complete, and, in addition, the phenolic compound contents remained stable. The proposed valorization procedure for WWP would allow an integral use of the pomace and the preservation of the phenolic compounds. Therefore, the stabilization process of the WWP would include a thermal blanching of fresh pomace, grinding, vacuum-packaging, and HHP (600 MPa/5 min), and this process would allow for obtaining an ingredient (valorized pomace, VP) rich in phenolic compounds, which could be used for the manufacturing of food products. 

The PCC of our valorized pomace was 777 mg 100 g^−1^ WB or 2489 mg 100 g^−1^ DB. It is difficult to compare the PCC of this valorized pomace with previous studies since most of them evaluate red grape pomace, and the preparation of the sample differs. Urquiaga et al. [30] reported higher PCC than ours in red wine pomace flour of 4111 mg 100 g^−1^ (DW), while Selani et al. [31] and Abdelhakan et al. [32] found lower values than ours in red grape varieties (784–942 mg 100 g^−1^ DW in dried seeds and peels; and 492 mg 100 g^−1^ DW in flour from peels, respectively).

The composition of the VP ingredient used for the preparation of burgers is shown in Table 2. The composition of VP depends on a multitude of factors, such as the grape variety, planting environment, or processing method [33]. The pH and aw were similar to those observed in WWP from other local grape varieties [14]. The moisture was higher than that obtained by these same authors, although, in the literature, we have found some data similar to ours [34] in grape marc waste.

Fat content presented values within the range offered by Antonić et al. [33] and Ayuso et al. [14]. However, the protein content was lower than previous studies in the literature [8,14,33]. Grape pomace represents a rich source of dietary fiber, and in addition, the transport of dietary antioxidants through the gastrointestinal tract may be an essential function of this dietary fiber [30]. Dietary fiber values were within the range reported by Antonic et al. [33].

In general, fiber increases the water-holding capacity, cooking yield, and juiciness in foods by forming gel networks with water absorption [35]. The content of fiber is a variable parameter since it depends on the proportion of branches included in the pomace. In addition, the fatty acid profiles showed high levels of unsaturated fatty acids, like w-3 (C18:3) and w-6 (C18:2), which are essential due to their high nutritional value and have their origin in grape seeds [7,33].

The determination of phenolic compound profiles is of particular interest for recognizing a possible relationship between the content of bioactive compounds and the antioxidant properties of VP. These data may provide valuable information for the characterization of the ingredient and also increase the economic value of the product. Many researchers have discovered that WWP is an excellent source of flavanols, flavonols, and phenolic acids [36,37]; however, most of them have evaluated red wine pomace. In the valorized cv., Cayetana grape pomace were identified and quantified catechins as (+)-catechin, (-)-epicatechin, galocatechin, epigallocatechin, and procyanidins B1 and B2; flavonols such as 3-O-galactoside and 3-O-glucoside are derivatives of kaempferol, quercetin, isorhamnetin, and myricetin. Caffeoyl, p-coumaroyl, and feruloyl acid and their tartaric esters, such as p-coumaroyltartaric (coutaric) and feruloyltartaric (fertaric), gallic and syringic acid, and stilbenes (resveratrol) (Table 2). When Jara–Palacios et al. [38] investigated the phenolic composition of grape pomace of the white variety Zalema, D.O., “Condado de Huelva” (Spain), they found flavanols such as catechin, epicatechin, and procyanidins B1, B2, and B3, besides procyanidin trime and tetramer. As flavonols, they reported rutinoside, galactoside and glucuronide of quercetine and glucuronide and the glucoside of kaempferol. Finally, gallic, protocatechuic, caftaric, caffeic, and p-coumaric acids were identified and quantified as phenolic acids. 

Since this is a white cultivar, it did not include anthocyanins. Procyanidins were the major group and accounted for 37.2% of total polyphenols, and catechins reached for 25.3% of total polyphenols. Thus, the most abundant phenolic compounds were the flavanols (62.5% of total polyphenols), while the flavonols were 33.0%; phenolic acids and stilbenes were present in lower amounts (6.2% and less than 1%, respectively, of the total polyphenols). This distribution is similar to that found in Jara-Palacios et al. [38] in their work previously cited. These researchers found percentages of 65%, 30%, and 4% of phenolic acids in the grape pomace of the cv. Zalema investigated. Previous studies on white pomace [37,39,40] found large variabilities comprising all individual phenolic compounds depending on the cultivar and maturity level, vintage status, climate, tissue parts, and winemaking techniques. However, besides the lack of anthocyanins in white grape pomace, no principal differences between red and white grape varieties were observed. In this sense, and to the best of our knowledge, this is the first time that data on the phenolic composition of white pomace treated by HHP has been provided. 

### 3.2. Effect of Valorized Pomace for the Preservation of Pork Burgers

The proximate composition (g 100 g^−1^) of burgers was, for moisture: 67.4 ± 1.1, protein: 19.5 ± 0.4, and fat: 9.0 ± 0.7. Moisture and protein values were similar to those found in the literature on pork burgers [41], although the fat value was lower than those reported by these authors. 

Burgers with VP could not be considered fiber-enriched foods because the level is lower than 6 g of fiber per 100 g of burger, which is required by European legislation (CE No 1924/2006) [42]. The calculated level of fiber in the burgers would be 0.24 g, 0.18 g, and 0.09 g per 100 g of burger with 3, 1, and 0.5% of VP, which are much lower than the levels required. However, in this particular case, the objective would not be to increase fiber levels but to obtain a substitute of additives like sulfite due to the incorporation of bioactive compounds in the burger from the pomace.

The pH of the burgers was affected by the addition of metabisulfite and the different levels of VP (Table 3). The control burgers had a higher pH than the other formulations (*p* < 0.001), although the differences are within the range of the second decimal of the measurements, so the effect could not be relevant for meat preservation. Moreover, there was a reduction in pH values as a function of increasing the levels of VP as compared to the control treatment. In burgers manufactured with VP, the decrease in pH was expected due to the low pH of the pomace (3.96), and therefore, it could be caused by the incorporation of organic acids from the pomace [8]. Similarly, the pH of beef burgers was reduced from 5.7 to 5.5 with the addition of grape pomace extract [32] and from 5.83 to 5.74 and to 5.68 with the addition of 2 and 4% of red grape pomace/seed powder [35]. In our case, the reductions of pH were not as marked as in those previous studies because VP was not dried as in those latter studies, so the effect of the changes in pH on other parameters like microbiology could be less significant.

The microbiological analysis of the burgers showed the absence of pathogens (*L. monocytogenes* and *Salmonella* spp. in 25 g of meat) at the beginning of storage, so they were not again analyzed at the end of storage. Initial levels of total mesophilic aerobic counts in the vacuum-packed formulations of burgers stored at 5 °C ranged between 6.2 and 6.6 log CFU g^−1^ (Table 3). Generally, the initial counts of the microorganisms tested were not affected by the addition of VP since no significant differences were observed with the control or MTB burgers. The total mesophilic aerobic bacteria count is generally used as an indicator of microbial spoilage, and according to general recommendations, the modifications in the meat quality become apparent to consumers when the microbial counts reach seven log CFU g^−1^ [43]. A possible explanation for this relatively high initial count levels in burgers may be attributed to the mincing process, which contributes to the total viable counts, likely as a consequence of the disruption of muscle structure, making nutrients easily available to microorganisms [44]. The initial counts were similar in all formulations and ranged between 6.0–6.7 log CFU g^−1^ for psychrophilics, 4.2–4.5 log CFU g^−1^ for molds and yeasts, 2.1–2.3 log CFU g^−1^ for *S. aureus*, 3.9–3.7 log CFU g^−1^ for total coliforms, and <1 log CFU g^−1^ for sulfite-reducing bacteria and *E. coli*. Several factors have been identified that could limit the antimicrobial activity of phenolic compounds when applied in meat systems. For example, the hydrophobic nature of phenolic compounds facilitates their accumulation in fat, which could make it difficult for contact between phenolic compounds and pathogenic microorganisms that accumulate in the hydrophilic phase of meat. In addition, proteins create strong complexes with phenolic compounds, and this could reduce their antimicrobial capacity [45].

As might be expected, microbial counts increased for all vacuum-packaged burgers during refrigerated storage, above the recommended values. For burgers to be commercialized, according to European regulations (Commission Regulation (EC) No 2073/2005, concerning food microbiology) [46], total viable counts must be less than 5.6–6.7 log CFU g^−1^, *E. coli* counts between 500 (m) and 10^3^ (M) CFU g^−1^ and *Salmonella* must be absent in 25 g [2]. Therefore, after 7 days of storage, all burgers would be above the recommended limits. Microbial spoilage is the main factor limiting the shelf-life of refrigerated meat products and is even more relevant in burgers because grinding and mixing increase the risk of contamination and reduce microbial stability [47]. Total viable counts of seven log CFU g^−1^ or higher are often indicative of sensory spoilage in meat [2]. In this case, due to the high initial microbial counts, a reduction in the storage temperature would be recommendable to reach a longer shelf-life.

Only the counts of molds and yeasts remained stable in the control burgers and those formulated with VP at the end of storage. After 7 days of storage, no differences in mesophilic and psychrophilic counts were observed between the different formulations. In contrast, total coliform counts were higher in the control than in the other formulations (*p* < 0.01), so in this case, VP and metabisulfite would have an effect against this group. 

The antimicrobial properties of sulfites are widely known, although the mechanisms by which sulfite inhibits microbial multiplication are very complex. The bacteria growth is inhibited more effectively than yeasts and molds, and yeasts are more resistant to the action of sulfites than bacteria or fungi [4]. In fact, sulfites have a broad spectrum of activity against most of the Gram-negative and several Gram-positive bacteria [5], and they may affect the synthesis and energy production of the bacterial cell, its DNA replication, and its membrane functions [3]. However, at the end of storage, unexpectedly, the utilization of metabisulfite had a negative effect on microbial counts of molds and yeasts and *S. aureus*, as they were higher than the control (*p* < 0.001 and *p* < 0.01, respectively). Only the total coliform counts were lower than the control (*p* < 0.01). The utilization of VP had a slight effect on microbial counts as it presented a similar effect as the metabisulfite for the control of total coliform counts during storage. In contrast, Belles et al. [3] observed a strong antimicrobial effect of sulfite in lamb burgers stored for 8 days, and Serrano and Bañón [2] found that the addition of 450 mg kg^−1^ of SO_2_ improved the microbial quality of fresh pork burgers, extending their shelf life for up to 21 days. The lack of effect of sulfites on microorganisms in our study is an unexpected result that is difficult to explain. A possible cause could be associated with the high initial level of counts in our burgers compared to those studies. Moreover, sulfites are often used in combination with ascorbic acid because they may inhibit its oxidation through the formation of hydroxysulfonate of dehydroascorbic acid [48]. The oxidation of MTB during the mixing process of the burgers could probably explain the lack of antimicrobial effect of this additive in our study. In fact, sulfites are chemically highly reactive additives and interact with a lot of meat components by nucleophilic addition or substitution mechanism [5]. As a result, they could have reduced activity in some meat products.

Pomace has an important antimicrobial activity, and their addition to food products decreases microbial growth [8]; however, at low concentrations of grape extracts (lower than 0.2%), it had no effect on the final population or only produced slightly lower final counts [49,50]. The lack of antimicrobial effect was explained by the low level applied and to matrix–phenol interactions that may limit the antimicrobial capacity of phenolic compounds. In the current study, the addition of VP to pork burgers avoided the increase in coliforms. The effect of pomace against coliforms was demonstrated in other studies [51,52].

In general, the formulation type of the burgers did not affect the color coordinates (Table 4). The lightness (CIE L*), redness (CIE a*), and yellowness (CIE b*) did not vary due to the formulation of the burger (Control, MTB, VP-0.5, VP-1, VP-3) since no significant differences were found because of the use of MTB or VP. The lack of changes in the appearance of burgers after the addition of VP would be positive to avoid the rejection of consumers at the purchase moment.

After 7 days of storage, lightness did not show changes in most burgers (except for the increase in VP-3). In contrast, the redness decreased significantly with storage time in the control and VP groups, while MTB showed a less intense reduction. The percentage of reduction in CIE a* on day one with respect to day seven was, for the control: −63%, MTB: −15%, VP-0.5: −39%, VP-1: −66%, and VP-3: −42%. Therefore, after 7 days of storage, CIE a* values were the lowest in the control and VP burgers with respect to the MTB batch. CIE b* showed the same trend as CIE a* during storage, with intense reductions in all groups except in MTB burgers. Chroma showed similar behavior, such as redness and yellowness. The hue angle was the only parameter affected by the incorporation of grape pomace, and on day one, it showed the highest values when burgers were prepared with VP-3. During storage, Hue increased in all groups, although on day seven, MTB showed significantly lower values compared to the others. 

Sodium metabisulfite was able to protect the meat from discoloration, keeping the color of the burgers stable during storage. Similarly, Belles et al. [3] also identified a protective effect of sulfite against the discoloration of lamb burger meat from 3 days post-packaging. Consumers associate a bright red color with freshness and superior meat quality, so darkening meat after 7 days of storage could cause consumer rejection. The color stability of sulfite added to burgers may be related to the ability of this compound to indirectly stabilize oxygen binding forms, thereby lessening the formation of metmyoglobin and providing a fresh appearance to red meat [5]. In contrast to the effect provided by the sulfites, VP did not reduce the discoloration in burgers during storage.

The initial lipid oxidation level ranged from 0.1 to 0.3 mg MDA kg^−1^ (*p* < 0.001) in the five burger formulations (Table 5). The TBA-RS values were higher in the control and MTB burgers than in burgers with VP (*p* < 0.001). After 7 days of storage, the burgers manufactured with VP showed the lowest lipid oxidation values, while the highest values were obtained in the MTB burgers, and control burgers showed intermediate values (*p* < 0.001). Differences in lipid oxidation development could be due to the positive effect of grape polyphenol compounds for meat preservation. Grape polyphenols have proved to have antioxidant effects [1,8], and they could limit lipid oxidation reactions. Phenolic compounds exert their antioxidant potential by scavenger-free radicals and inhibiting enzymes responsible for their formation, chelating metallic ions, protecting antioxidants such as ascorbic acid and vitamin E, and activating antioxidant enzymes such as catalase and superoxide dismutase [6,53]. Similar results were found in beef and salmon burgers with red grape pomace powder [6,32]. Moreover, the addition of sulfite did not inhibit the propagation of oxidative reactions compared to the control, although sulfites also have an antioxidant effect on meat products [5].

During storage, the lipid oxidation values decreased unexpectedly in the control samples (*p* < 0.000), while it remained stable in the other groups. Similar to us, Nardoia et al. [10] observed a decrease in TBARS values during the storage of chicken burgers, presumably due to intermolecular reactions in the malonaldehyde formed (polymerization) and reactions with other constituents, especially amino acids/proteins. TBA-RS value (malonaldehyde kg^−1^) is used as an index for measuring oxidative rancidity, which takes place in meat products during storage. Lipid oxidation is one of the main limiting factors for the quality and acceptability of meat and meat products. However, in vacuum-packaged products and at short times of storage, as in this case, its increase would be limited. Normally, lipid oxidation development is associated with discoloration changes during storage [32]; however, in the current study, despite the low levels of lipid oxidation of burgers with WWP, they presented an important discoloration during storage. Changes in color in these burgers could be explained by microbial spoilage in burgers, as microbial contamination is related to undesirable discoloration of meat [54,55] because microbial growth contributes to the oxidation of myoglobin [56].

Protein oxidation showed a similar pattern as lipid oxidation since initially, the highest value of protein oxidation was found in the control group while the lowest was found at VP-0.5; the other groups showed intermediate values (*p* < 0.05) (Table 5). Protein oxidation is detrimental to the overall quality of fresh meat and meat products. Lipid oxidation products and free radicals promote protein oxidation, leading to chemical changes in proteins that decrease protein solubility and prevent natural proteolysis, which negatively affects the organoleptic characteristics of the meat, such as tenderness and juiciness [57,58]. After storage, no significant differences were observed between the different formulations. In contrast, Andres et al. [11] observed a lower intensity of protein oxidation processes in lamb burgers manufactured with red grape pomace extract stored for 7 days in modified atmospheres, and similarly, Jongberg et al. [59] observed that white grape extract reduced the cross-link formation in beef patties stored in high oxygen modified atmospheres. 

The total phenolic compound content of MTB and VP-3 burgers was higher than in the control burgers, while burgers with VP-0.5 and VP-1 showed intermediate values (*p* < 0.001) (Table 5). The phenolic compound content of burgers was maintained after storage or even unexpectedly increased in the control burgers, and on day seven, no differences were found among the groups. The high stability of phenolic compounds in burgers during storage was in line with the great oxidative stability of burgers during storage.

Values of phenolic compounds content in the control burgers were lower than those reported in beef burgers by Ahmed et al. [60], with 152 mg GAE 100 g^−1^ in burgers with a formulation based on chickpea powder, black and white pepper, garlic powder, and onion powder. In the current burgers, the PCC could be explained by the addition of black pepper, garlic powder, and onion powder, as in the previous study. However, surprisingly, the highest values of phenolic compounds were observed in MTB burgers. A possible explanation could be that sulfite has been reported to be a substance that potentially interferes with the total phenolic content determination by the Folin–Ciocalteu method [61,62]. Therefore, the levels of phenolic compounds in this group could be overestimated. 

Among the burgers manufactured with the ingredient, the highest values of phenolic compounds were found in burgers with the highest levels of VP (3%), which would be explained by the phenolic compounds present in the pomace. On the other hand, burgers with the VP showed higher levels of phenolic compounds than in the control group. According to Table 2, the total of phenolic compounds in VP would be 766.7 mg 100 g ^−1^, and as a consequence, in a 100 g burger, the levels of phenolic compounds with an origin in the pomace would be approximately 3.8 mg (VP-0.5), 7.6 mg (VP-1), and 23.0 mg (VP-3). These levels were proportional to the increases detected by the Folin method in burgers. However, this method has limitations, like interferences between polyphenols and other compounds like amino acids and sugars, which have also been previously reported in meat products [63]. This could explain the increase in phenolic compound values in the control burgers after storage since, during this time, proteolytic reactions could appear, and increases in free amino acids (which could have phenolic groups) are expected.

**Figure 1:**
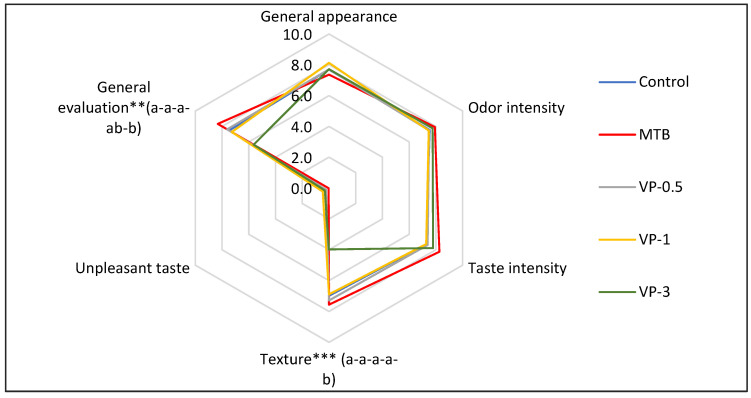
Sensory evaluation of different cooked burgers. ** *p* < 0.01 *** *p* < 0.001. Different letters (a-b) indicate significant differences in the Tukey test. Control (burgers manufactured without sodium metabisulphite); MTB (burgers manufactured with sodium metabisulphite); VP-0.5 (burgers manufactured with 0.5% (*w*/*w*) valorized white wine pomace); VP-1 (burgers manufactured with 1% (*w*/*w*) valorized white wine pomace); VP-3 (burgers manufactured with 3% (*w*/*w*) valorized white wine pomace).

In general, the addition of VP did not affect the sensory parameters of freshly grilled burgers on day one (Figure 1). Differences were only observed in the texture and overall evaluation of the burgers due to the incorporation of wine pomace. The panelists indicated lower texture acceptance values in the VP-3 burgers (*p* < 0.000), while the other treatments showed similar values among them. The highest values for the general evaluation were observed in the control, MTB, and VP-0.5, which did not differ from each other, and they were higher than those presented by VP-1 and VP-3 (*p* < 0.001). A panelist commented that the pomace was perceptible when chewing. In contrast, De Alencar et al. [1] did observe an effect in the addition of grape skin flour in the formulation of beef burgers. These authors associated the differences found in texture with the higher fiber content of those formulations in addition to the lower fat content in burgers with higher levels of grape skin flour. In our case, the seeds were included in the ingredients of the pomace, and this part was difficult to grind, so this process should be improved to avoid the rejection of the ingredients added to the burgers.

In general, one of the main concerns regarding the use of agri-food by-products, like pomace, is the negative effects on the organoleptic properties of the applied foods. Thus, in line with our results, high amounts of grape pomace flour added to salmon burgers generally achieved low scores for flavor and texture attributes [64]. For that reason, pomace-derived products have to be incorporated at low doses so that they can have reduced activity.

Appearance and odor are the primary sensory attributes of meat products that influence their perception and the choice of consumers [65]. In our study, the general appearance was similar in all treatments in line with the lack of differences in instrumental color parameters (CIE L*a* and b*) on day one in the fresh burgers. However, it should be noted that the sensory test was performed on the cooked burgers, while instrumental color was measured on the fresh burgers.

## 4. Conclusions

In this study, white wine pomace was valorized by the application of thermal blanching before processing by hydrostatic high pressure; as a result, an ingredient rich in fiber and phenolic compound content was obtained. This valorization process does not need solvents and allows for the integral use of the by-product (no residues are generated). No previous studies have evaluated the effect of valorized pomace on meat products. The valorized pomace could be added at low levels (maximum 3%) for the preparation of burgers due to its strong taste. The ingredient developed prevented the progress of lipid and protein oxidation during manufacturing and the refrigerated storage of burgers, but it presented a limited antimicrobial effect (only for coliforms) and did not avoid meat discoloration. Due to the low increase in oxidation in the vacuum-packaged burgers during refrigeration, the antioxidant effect of valorized pomace was not notable; however, maybe in dry-cured meat products, which need a long maturation time, its inclusion could be effective. In contrast, metabisulfite prevented discoloration during storage and, at a microbiological level, only reduced the coliform count. Future studies should evaluate whether the utilization of red grape pomace valorized by this method produces a similar effect as the white one or if it avoids the decoloration of the burgers due to its anthocyanin pigments. In that case, a mixture of red and white grape pomace could be suitable for the preservation of fresh meat products like burgers.

## Figures and Tables

**Table 1:** Changes in the polyphenoloxidase (PPO) enzyme activity, the total phenolic compounds content (mg GAE, 100 g^−1^) and microbial counts (log CFU g^−1^) during the process of manufacture of the valorized ingredient from white wine pomace.

	Initial-WWP	TB	TB+HHP	*p*-Value Initial-TB	*p*-Value TB-HHP	*p*-Value Initial-HHP
PPO (%)	100.0a ± 1.6	0.9b ± 0.0	1.3b ± 0.0	***	ns	***
Phenolic compounds	457.0b ± 24.6	758.2a ± 30.2	766.7a ± 15.6	**	ns	***
						
Mesophilic counts	5.8a ± 0.1	4.5ab ± 1.2	3.3b ± 0.2	ns	ns	**
Molds and Yeasts	4.6a ± 0.1	2.7b ± 0.3	1.4c ± 0.5	***	**	***
Enterobacteria	2.2 ± 1.1	<1	<1	ns	ns	ns

Initial-WWP: White wine pomace (initial); TB: Thermal blanching, thermally treated pomace; TB+HHP: thermally treated and high pressure treated pomace. *p*-value initial-TB (Student’s *t*-test between initial purée and TB purée); *p*-value TB-HHP (Student’s *t*-test between TB purée and HHP purée); *p*-value initial-HHP (Student’s *t*-test between initial purée and TB+HHP purée). ns. (non-significant differences). ** *p* < 0.01 *** *p* < 0.001. Different letters in the same row indicate significant differences in the Tukey test.

**Table 2:** Proximate composition (g 100 g^−1^), pH, aw, fatty acid methyl esters composition (%) and individual phenolic compounds profile (%) of valorized pomace (thermally blanched, milling and high pressure treated).

	Valorized Pomace
pH	3.96 ± 0.03
Aw	0.971 ± 0.013
**Proximate composition (g 100 g^−1^)**	
Moisture	69.2 ± 1.5
Fiber	18.0 ± 0.9
Protein	2.3 ± 0.2
Fat	1.7 ± 0.1
**Fatty acids profile (%)**	
C12:0	0.0 ± 0.0
C14:0	0.1 ± 0.0
C16:0	9.6 ± 0.1
C16:1	0.2 ± 0.0
C17:1	0.1 ± 0.0
C17:1	0.0 ± 0.0
C18:0	4.8 ± 0.1
C18:1	19.7 ± 0.1
C18:2	63.2 ± 0.3
C18:3	1.8 ± 0.0
C20:0	0.3 ± 0.0
C20:1	0.2 ± 0.0
**Individual phenolic compounds content (%)**	
***Flavanols***	
PB1+PB3	31.62 ± 1.10
PB2	5.59 ± 0.14
*Total procyanidins*	**37.21** ± 1.24
(+)-Catechin	15.70 ± 1.24
(-)-Epicatechin	8.27 ± 0.69
Epigallocatechin	1.31 ± 0.20
*Total Catechin*	**25.27** ± 2.13
**Total Flavanols**	**62.48** ± 3.37
***Flavonols***	
Myricetin-3-galactoside	0.05 ± 0.01
Myricetin-3-glucoside	0.10 ± 0.01
Quercetin-3-glucuronide	18.60 ± 0.60
Quercetin-3-glucoside	9.93 ± 0.25
Kaempferol- 3-glucoside+3-rutinoside	4.12 ± 0.11
Isorhamnetin-3- glucoside+-3-rutinoside	0.25 ± 0.02
**Total Flavonols**	**33.05** ± 0.99
***Acids and derivatives***	
Gallic	0.79 ± 0.05
Syringic	1.87 ± 0.58
Coutaric	0.70 ± 0.03
Caffeic	0.07 ± 0.00
Ferouyl-tartaric	0.83 ± 0.02
Coumaric	0.08 ± 0.00
Ferulic	0.11 ± 0.70
**Total Acids and derivatives**	**4.46** ± 0.70
***Stilbenes***	
trans -Resveratrol	0.01 ± 0.00

**Table 3:** pH and microbiological changes (log colony forming units, CFU g^−1^) of the burgers manufactured with VP.

	Storage (Days)	Control	MTB	VP-0.5	VP-1	VP-3	*p*-Value
pH		5.86a ± 0.01	5.84b ± 0.00	5.84b ± 0.01	5.83b ± 0.01	5.80c ± 0.02	***
Mesophilic counts	*1*	6.5 ± 0.0	6.3 ± 0.1	6.2 ± 0.4	6.4 ± 0.2	6.6 ± 0.1	*ns*
	*7*	8.1 ± 0.5	7.8 ± 1.7	8.1 ± 0.3	7.8 ± 0.2	8.2 ± 0.4	*ns*
	*P-storage*	***	*ns*	***	***	***	
Psychrophilic counts	*1*	6.6a ± 0.1	6.0b ± 0.3	6.7a ± 0.5	6.5ab ± 0.4	6.6a ± 0.1	*
	*7*	8.0 ± 0.6	8.0 ± 0.5	7.9 ± 0.4	7.7 ± 0.5	8.5 ± 0.5	*ns*
	*P-storage*	**	***	**	**	***	
Molds and Yeasts	*1*	4.5 ± 0.2	4.4 ± 0.3	4.2 ± 0.3	4.4 ± 0.2	4.5 ± 0.2	*ns*
	*7*	4.8b ± 0.2	5.4a ± 0.3	4.4b ± 0.4	4.6b ± 0.2	4.5b ± 0.3	***
	*P-storage*	*ns*	***	*ns*	*ns*	*ns*	
*S. aureus*	*1*	2.1 ± 0.2	2.3 ± 0.3	2.0 ± 0.1	2.1 ± 0.3	2.2 ± 0.3	*ns*
	*7*	2.1b ± 0.2	2.6a ± 0.1	2.3b ± 0.3	2.1b ± 0.3	2.0b ± 0.2	**
	*P-storage*	*ns*	*	*	*ns*	*ns*	
Sulfite-reducing bacteria	*1*	<1	<1	<1	<1	<1	*ns*
	*7*	<1	<1	<1	<1	<1	*ns*
	*P-storage*	*ns*	*ns*	*ns*	*ns*	*ns*	
Total coliforms	*1*	3.7 ± 0.1	3.9 ± 0.3	3.9 ± 0.1	3.7 ± 0.2	3.9 ± 0.1	*ns*
	*7*	5.1a ± 0.1	4.3b ± 0.3	4.7b ± 0.2	4.6b ± 0.3	4.7b ± 0.3	**
	*P-storage*	***	*ns*	***	**	***	
*E. coli*	*1*	<1	<1	<1	<1	<1	*ns*
	*7*	<1	<1	<1	<1	<1	*ns*
	*P-storage*	*ns*	*ns*	*ns*	*ns*	*ns*	

Control (burgers manufactured without sodium metabisulfite); MTB (burgers manufactured with sodium metabisulfite); VP-0.5 (burgers manufactured with 0.5% (*w*/*w*) valorized white wine pomace); VP-1 (burgers manufactured with 1% (*w*/*w*) valorized white wine pomace); VP-3 (burgers manufactured with 3% (*w*/*w*) valorized white wine pomace). ns. (non-significant differences). * *p* < 0.05 ** *p* < 0.01 *** *p* < 0.001. Different letters in the same row indicate significant differences in the Tukey test.

**Table 4:** Instrumental color changes in the burgers manufactured with VP.

	Storage (Days)	Control	MTB	VP-0.5	VP-1	VP-3	*p*-Value
CIE L*	1	54.4 ± 1.7	54.3 ± 1.1	53.8 ± 1.7	53.0 ± 1.4	52.3 ± 0.9	*ns*
	7	54.6 ± 1.3	54.7 ± 1.9	54.4 ± 1.1	54.3 ± 0.5	55.7 ± 1.0	*ns*
	*P-storage*	*ns*	*ns*	*ns*	*ns*	***	
CIE a*	1	11.1 ± 0.7	11.9 ± 1.8	10.9 ± 0.9	10.9 ± 0.8	10.0 ± 0.4	*ns*
	7	4.1c ± 1.6	10.1a ± 1.9	6.6bc ± 1.2	7.2b ± 1.4	5.8bc ± 0.8	***
	*P-storage*	***	*ns*	***	**	***	
CIE b*	1	13.1 ± 0.7	12.9 ± 1.4	12.7 ± 0.4	13.5 ± 0.4	14.0 ± 0.6	*ns*
	7	10.4b ± 0.8	12.9a ± 1.6	11.4ab ± 0.4	11.9ab ± 0.9	12.6a ± 0.4	**
	*P-storage*	***	*ns*	**	**	**	
Chroma	1	17.2 ± 0.9	17.5 ± 2.1	16.8 ± 0.7	17.4 ± 0.7	17.2 ± 0.6	*ns*
	7	11.3c ± 0.5	16.4a ± 2.4	13.3b ± 0.8	14.0b ± 1.0	13.9b ± 0.7	***
	*P-storage*	***	*ns*	***	***	***	
Hue	1	49.7b ± 1.7	47.6b ± 2.4	49.4b ± 2.0	51.0b ± 1.9	54.3a ± 1.5	***
	7	68.8a ± 9.0	52.2c ± 1.9	60.9abc ± 3.8	58.9bc ± 5.5	65.5ab ± 2.3	**
	*P-storage*	**	**	***	*	***	

Control (burgers manufactured without sodium metabisulfite); MTB (burgers manufactured with sodium metabisulfite); VP-0.5 (burgers manufactured with 0.5% (*w*/*w*) valorized white wine pomace); VP-1 (burgers manufactured with 1% (*w*/*w*) valorized white wine pomace); VP-3 (burgers manufactured with 3% (*w*/*w*) valorized white wine pomace). ns. (non-significant differences). * *p* < 0.05 ** *p* < 0.01 *** *p* < 0.001. Different letters in the same row indicate significant differences in the Tukey test.

**Table 5:** Lipid (mg MDA kg^−1^) and protein oxidation (nmols carbonyls mg protein^−1^) changes and phenolic compounds content (mg 100 g^−1^) of the burgers manufactured with VP.

	Storage (Days)	Control	MTB	VP-0.5	VP-1	VP-3	*p*-Value
Lipid oxidation	1	0.282a ± 0.008	0.265a ± 0.072	0.124b ± 0.009	0.100b ± 0.022	0.105b ± 0.006	***
	7	0.193b ± 0.029	0.261a ± 0.011	0.108c ± 0.009	0.097c ± 0.003	0.110c ± 0.004	***
	*P-storage*	***	*ns*	*	*ns*	*ns*	
Protein oxidation	1	2.231a ± 0.447	1.909ab ± 0.189	1.575b ± 0.465	2.033ab ± 0.171	1.832ab ± 0.141	*
	7	1.896 ± 0.230	1.603 ± 0.290	1.429 ± 0.299	1.347 ± 0.305	1.371 ± 0.754	*ns*
	*P-storage*	*ns*	*ns*	*ns*	**	*ns*	
Phenolic compounds	1	59.86c ± 3.06	72.71a ± 3.11	65.01bc ± 3.88	64.89bc ± 3.11	67.67b ± 2.00	***
	7	64.48 ± 2.39	77.23 ± 18.02	63.53 ± 3.39	59.94 ± 7.07	64.90 ± 10.57	*ns*
	*P-storage*	*	*ns*	*ns*	*ns*	*ns*	

Control (burgers manufactured without sodium metabisulphite); MTB (burgers manufactured with sodium metabisulphite); VP-0.5 (burgers manufactured with 0.5% (*w*/*w*) valorized white wine pomace); VP-1 (burgers manufactured with 1% (*w*/*w*) valorized white wine pomace); VP-3 (burgers manufactured with 3% (*w*/*w*) valorized white wine pomace). ns. (non-significant differences). * *p* < 0.05 ** *p* < 0.01 *** *p* < 0.001. Different letters in the same row indicate significant differences in the Tukey test.

## Data Availability

The authors confirm that the data supporting the findings of this study are available within the article and the raw data supporting the findings are available from the corresponding author, upon reasonable request.
